# Outcomes of ovarian transposition in cervical cancer; an updated meta-analysis

**DOI:** 10.1186/s12905-022-01887-8

**Published:** 2022-07-22

**Authors:** Alexandros Laios, Mohamed Otify, Argyro Papadopoulou, Ioannis D. Gallos, Thomas Ind

**Affiliations:** 1 Department of Gynaecological Oncology, St James’s University Hospital, Leeds Teaching Hospitals NHS Trust, Beckett Street, Harehills, Leeds, LS97TF UK; 2 Department of Gynaecologic Oncology, Liverpool Women’s NHS Foundation Trust, Crown Street, Liverpool, L8 7SS UK; 3 Tommy’s National Centre for Miscarriage Research, Institute of Metabolism and Systems Research (IMSR), University of Birmingham, Birmingham Women’s Foundation NHS Trust, Heritage Building, Mindelsohn Way, Edgbaston, Birmingham, B15 2TH UK; 4 Department of Gynaecological Oncology, Royal Marsden Hospital, Fulham Road, London, SW36JJ UK; 5 St Georges’s University of London, Blackshaw Road, London, SW170QT UK

**Keywords:** Cervical cancer, Ovarian transposition, Ovarian preservation, Ovarian metastases, Ovarian cysts

## Abstract

**Background:**

Cervical cancer is the most common indication for ovarian transposition in reproductive-age women. Ovarian transposition should be performed in premenopausal women undergoing pelvic irradiation to preserve ovarian function, and prevent early menopause. As women become more knowledgeable about their fertility options, it is still unclear who will benefit from the intervention. We updated our previous meta-analysis of ovarian function preservation, symptomatic ovarian cysts, and metastases to the transposed ovaries following ovarian transposition in cervical cancer patients to further guide current clinical practice.

**Methods:**

A systematic search of Medline, Embase, Web of Science, and The Cochrane Library databases, dating from January 1980 to July 2021, was conducted. We computed the summary proportions of women who had ovarian function preservation, non-ovarian cyst formation and metastases to the transposed ovaries following ovarian transposition by random-effects meta-analysis and we explored study heterogeneity by type of radiotherapy.

**Results:**

There were 29 publications reporting on 1160 women with cervical cancer who underwent ovarian transposition. In the group that underwent surgery alone, 91% of the women had preserved ovarian function (95% CI 83–100), 89% (95% CI 80–99) of women who did not develop ovarian cysts, and 99% (95% CI 1–5) of women who did not suffer metastases to the transposed ovaries. In the surgery ± brachytherapy (BR) group, the proportion of women with the preserved ovarian function was 93% (95% CI 76–113), 84% (95% CI 69–103) of women who did not develop ovarian cysts, and 99% (95% CI 82–120) of women who did not suffer metastases to the transposed ovaries. In the external beam pelvic radiotherapy (EBRT) ± BR ± surgery group, the proportion of women with the preserved ovarian function was 61% (95% CI 55–69), and 95% (95% CI 85–107) of women who developed ovarian cysts. There were no metastases to the transposed ovaries in that group.

**Conclusions:**

In women with cervical cancer, ovarian transposition offers a significant preservation of the ovarian function. Despite an expected incidence of ovarian cyst formation, it carries almost no risk for metastases to the transposed ovaries.

## Introduction

Globally, cervical cancer ranks fourth among female malignancies and represents a major global health challenge [1]. Nearly 40 percent of women with cervical cancer will be affected during their reproductive years, when they desire future fertility [2]. The focus of holistic cancer treatment has shifted to balancing oncological outcomes with reproductive benefits, and women are becoming increasingly aware of their reproductive choices [3]. Surgery for fertility preservation (FP) has become the standard of care for women with low-risk, early-stage disease of such preventable nature [4]. Nevertheless, the subject of fertility sparing treatment for cervical cancer remains a complex one. Offering FP treatments is not just about trachelectomy, but involves counselling, respecting patients’ prioritisations in outcomes, and considerations for approach and follow-up [5].

Cervical cancer is the most common indication for ovarian transposition (OT) in reproductive-age women. Amongst FP options, OT has now been established as a reliable and straightforward method with reduced morbidity [6]. Although the procedure has become minimally invasive, it can still delay definitive treatment, which can negatively affect outcomes [7]. In the case of cervical cancer, this procedure may be considered in young premenopausal women proceeding to pelvic radiotherapy (RT) [8]. Oocytes are uniquely sensitive to radiation injury, and doses as low as 10 Gy can trigger a premature ovarian failure [9]. In such patients receiving external beam radiotherapy (EBRT), the ovaries can be transposed laterally, above the pelvic brim, without tension on the vascular pedicle [10]. Standardized criteria for the preservation and transposition of the ovaries have been proposed [11].

Against the background expectation, OT remains paradoxically underused [12]. Previously, we published a systematic review of primary outcomes for OT in women with gynaecological cancers. Our meta-analysis of the reported studies published between 1980 and 2014 leveraged a significant association between OT and ovarian function preservation, but a negligible risk for metastases to the transposed ovaries, despite a common incidence of ovarian cysts [13]. The new ESGO guidelines for the management of patients with cervical cancer within a multidisciplinary setting have recently been released [8]. Earlier last year, the British Gynaecological Cancer Society (BGCS) released their guidelines for the diagnosis and management of cervical cancer. They acknowledged the scarcity of available data evaluating the OT for preserving ovarian function in cervical cancer patients notably due to (a) the small number of patients, (b) the wide variation in the type of transposition surgery performed, and (c) the absence of analysis on the impact from various postoperative treatments [14]. We hypothesized that the OT outcomes would differ between cervical cancers and other pelvic cancers due to different primary surgical procedures and radiotherapy fields. To further guide clinical practice, we aimed to update on our previous systematic review and meta-analysis of the proportions of women diagnosed with cervical cancer, who had their ovarian function preserved, and who did not develop symptomatic ovarian cysts and metastases to the transposed ovaries, following ovarian transposition.

## Materials and methods

### Studies identification

The population of interest included premenopausal women with a diagnosis of cervical cancer who might require RT with or without surgery. Patients who underwent reposition of the ovaries without the need for adjuvant RT, and who underwent unilateral ovary transposition were also included. Treatment involved OT, and outcomes included ovarian function preservation, metastatic ovarian cancer, and symptomatic or asymptomatic ovarian cysts. MEDLINE, EMBASE, Web of Science and The Cochrane Library were searched for articles published between January 1980 and July 2021. In our search, we combined text and terms from Medical Subjects with Emtree Headings: women OR female OR gynaecological malignancy OR gynaecological cancer OR cervical cancer OR cervical carcinoma AND ovarian transposition OR oophoropexy AND ovarian preservation OR fertility preservation OR fertility-sparing OR ovarian function OR premature ovarian failure OR ovarian cysts OR metastases.

As this was a systematic review, no ethical approval was required. The review was carried out according to the Preferred Reporting Items for Systematic Reviews and Meta-analyses (PRISMA) guidelines. As this was a secondary analysis, the methodology has already been described [12, 13].

### Outcomes of interest

The primary outcome was the ovarian function preservation following OT. Secondary outcomes included ovarian cyst formation and ovarian metastases. The following information was extracted; publication date and type of study, duration of follow-up, type of ovarian transposition, ovarian function preservation, and incidence of metastasis, ovarian cyst formation and related complications. Menopausal symptoms, serum FSH levels, E2 levels were primarily used to determine whether ovarian function was preserved. In the meta-analysis, only patients with follow-up data were included. The methodological Index for Non-Randomised Studies (MINORS), which assesses the quality of included studies, was implemented [15]. We reported our results in accordance with the guidelines of Meta-analysis of Observational Studies in Epidemiology (MOOSE) [16].

### Statistical analysis

In the absence of control groups, our analysis of outcomes involved calculating—instead of odd ratios—the proportion of women with preserved ovarian function, without ovarian cysts, and without metastases to transposed ovaries per total number of women undergoing OT. In other words, we performed a single-arm meta-analysis with effect estimates (probability). Although our outcomes were time dependent, data were not sufficient to calculate log hazard ratios from the individual studies. For each study, we calculated the logarithm of the ratio and its corresponding standard error. A random-effects model was used to perform a meta-analysis with inverse-variance weighting. For each outcome, forest plots were created showing individual study proportions with confidence intervals (CIs) and the overall pool estimate. Heterogeneity was assessed using the I^2^ test. Egger's weighted regression test was applied to funnel plot asymmetry. Statistics were analyzed using Stata 12.0 (Stata Corp, College Station, TX).

## Results

**Fig. 1:**
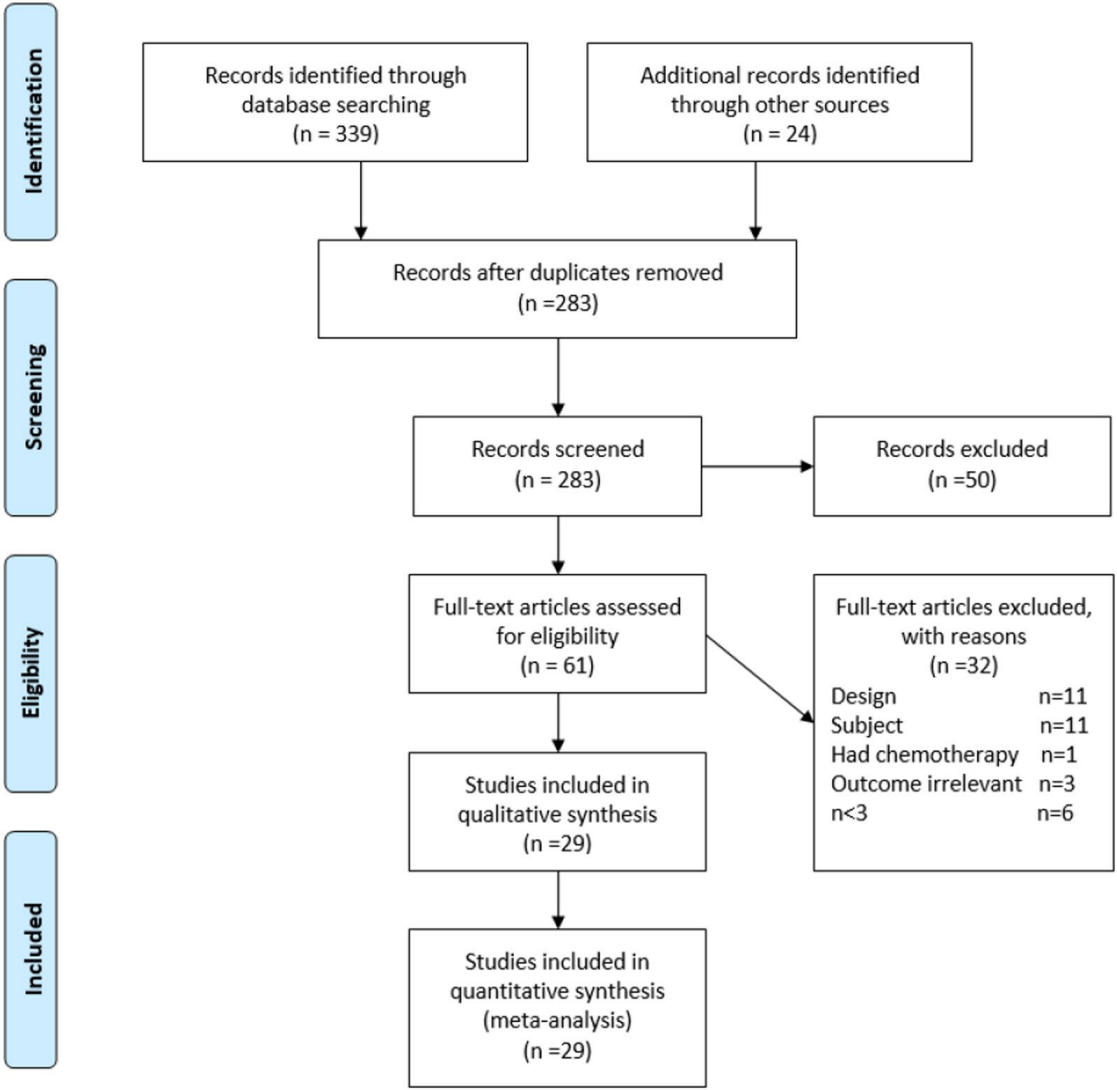
PRISMA 2009 flow diagram of our systematic search

The electronic search strategy initially yielded 363 citations, of which the first article was published in 1980. Figure 1 shows how the articles were selected for inclusion. Following initial screening and assessment of eligibility, 92 studies were extracted for full-text examination. A total of 29 primary studies, reporting on 1160 women with cervical cancer, who underwent OT, were included in this review. The primary study characteristics are shown in Table 1. As this was an update, an additional seven studies published from 2013 to date, including 441 patients were included in this review.

**Table 1:** Summary of the cervical cancer study characteristics

		Population and Intervention				Study groups			Outcomes		
First author, publication year	Study Design	Comments	Patients (N)	Type of ovarian transposition	Follow up (Median, range in months)	Surgery Only	Surgery + BR	Surgery + EBRT ± BR	Retained Function	Cyst	Metastasis
Hodel [36] 1982	Retrospective	Stage 1B cervical cancer	7	Open	NR	0	1	6	5	1	0
Husseinzadeh [17] 1984	Prospective	15/22 from surgery only group were included for which FSH levels were available	19	Open	NR	15	0	4	17	0	0
Ploch [18] 1988	Prospective	Focus on ovarian function only, additional analysis of location of transposed ovaries	22	Lap	13 (2–23)	5	5	12	15	0	0
Owens [19] 1989	Retrospective	All but three patients had early-stage cervical cancer, one patient had unilateral OT	14	Open	18	6	0	8	13	0	0
Chambers [21] 1990	Retrospective	Stage 1 cervical cancer, description of lateral OT	25	Open (sc)	14 (2–23)	25	0	0	22	6	0
Van Beurden [39] 1990	Retrospective	FSH levels were available for 6 patients	6	Open	23 (10–36)	0	0	6	1	0	0
Chambers [21] 1991	Retrospective	Stage 1 cervical cancer, ovarian preservation causally related to estimated scattered dose to ovaries	38	Open	35	24	0	14	27	7	0
Anderson [22] 1993	Retrospective	Comparison with a non- transposition group	82	Open / Lap	44	58	0	24	51	38	1
Bidzinski [23] 1993	Prospective	Stage 1 cervical cancer, ultrasound examination showed distinct reduction of transposed ovary echo structure in 91% cases	48	Open	41 (10–72)	9	24	15	45	0	0
Feeney [24] 1995	Retrospective	Ovarian function is reserved only in 50% of patients with postoperative BR	132	Lap	24	104	0	28	115	4	2
Clough [6] 1996	Prospective	Unilateral OT, success rate 100% in patients younger than 40 years old	17	Open	23.6 (12–33)	0	11	6	15	0	0
Covens [37] 1996	Retrospective	Patients with 1B cervical cancer prior to RT	3	Open	32	0	0	3	2	0	0
Fujiwara [25] 1997	Retrospective	Description of a new technique for OT	27	Open (sc)	27 (10–44)	25	1	1	26	18	0
Morice 1998	Prospective	Only 14/ 24 were included as they were repeated in other paper published by the same author	14	Lap	6	0	10	4	11	0	0
Morice [26] 2000	Prospective	95/107 patients were included; 12 patients were lost on follow up	95	Lap	31 (10–56)	11	59	25	79	25	1
Buekers [27] 2001	Retrospective	Stage 1 cervical cancer, 27 women had unilateral oophorectomy for intraoperative suspicion or vascular compromise	80	Open	87 (43–126)	54	0	26	64	0	0
Olejek [28] 2001	Prospective	Significance in ovarian preservation between RT and non-RT groups	44	Open	60	19	6	19	31	3	0
Yamamoto [11] 2001	Prospective	Regression analysis of risk factors for ovarian metastases	56	Open	12	30	0	26	50	0	0
Nagao [29] 2006	Retrospective	Subcutaneous fat OT, direct comparison to a non-transposition group	27	Open (sc)	65	22	0	5	25	3	0
Pahisa [30] 2008	Prospective	24/28 patients with 1b1 cervical cancer were included for which follow up was available	24	Open / Lap	44	13	6	5	20	2	0
Al-Badawi [31] 2010	Retrospective	Stage 1–2 cervical cancer, 11/14 women were < 40 years old	15	Lap	33	0	0	15	11	0	0
Han [38] 2011	Retrospective	19/29 patients with cervical cancer for which FSH was available	19	Open / Lap	17.2	0	0	19	11	3	0
Hwang [32] 2012	Retrospective	39/53 patients were included; 14 patients were lost on follow up or FSH not available	39	Open / Lap	39.8	8	0	31	18	1	0
Zhao [43] 2013	Retrospective	Stage 1–2 cervical cancer, risk factors for ovarian metastases reported	105	Open	N/R	105	0	0	-	-	2
Shou [40] 2015	Retrospective	6/26 patients with IIB-IIIB cervical cancer for which follow-up was available	26	Lap	N/R	0	0	26	18	-	0
Du Z [33] 2017	Retrospective	52/86 patients had concurrent chemotherapy; the relationship between ovarian function and ovarian limited dose (IMRT) in radiotherapy was evaluated	86	Open	6	21	0	13	34	-	-
Swift [34] 2018	Retrospective	9 patients with stage 1B1-2A cervical cancer, those who had chemoradiotherapy were excluded, description of a novel technique of laparoscopic lateral OT	6	lap	8–103	2	0	4	6	1	0
Hoeckman [35] 2018	Retrospective	23 patients with cervical carcinoma	23	Open/lap	34.5 (1.5–96)	0	0	23	16	0	0
Lv [41] 2019	Retrospective	77/150 patients,45 years who had complete follow-up, the association between the location of the transposed ovary and the ovarian dose was examined	77	Open	12	0	0	77	56	0	0
Yin L [42] 2019	Retrospective	105/118 patients received limited dose IMRT	118	Open	12	0	0	118	41	-	0
Total			1160		2–126	449	132	579	819	125	4

**Fig. 2:**
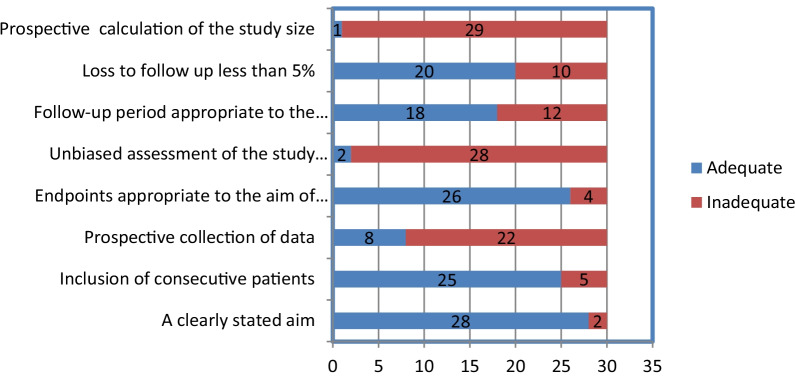
Quality assessment of the observational studies according to the MINORS criteria

Figure 2 shows the quality assessment of the studies in the MINORS checklist. The MINORS criteria score was 10.1 (range 5–13) out of a maximum score of 16. The studies were all observational. 7/24 (29.1%) of the studies included consecutive patients in 21/24 (87.5%). In 20/24 (83.3%) studies, outcomes were adequately defined. Study outcomes were not blinded and sample sizes were not calculated prospectively. 19/24 (79.1%) studies had a follow-up period longer than 12 months. Patients younger than 40 years of age were usually treated with this procedure in most studies. A number of surgical techniques were described, including laparotomy and minimally invasive surgery. In two studies, the ovaries were transposed to the subcutaneous tissue [9, 25].

Unilateral or bilateral OT was performed. A total of 449 patients had surgery alone in the form of radical hysterectomy (RH) ± pelvic lymphadenectomy (PLND) ± paraaortic lymph node dissection (PALND) (Group A); 132 patients had postoperative brachytherapy (BR) ± surgery (Group B); 579 patients had postoperative external beam radiotherapy (EBRT) ± BR ± surgery (Group C). Follow-up ranged from 2 to 126 months. The primary study characteristics are shown in Table 1.

### Preserved ovarian function

**Fig. 3:**
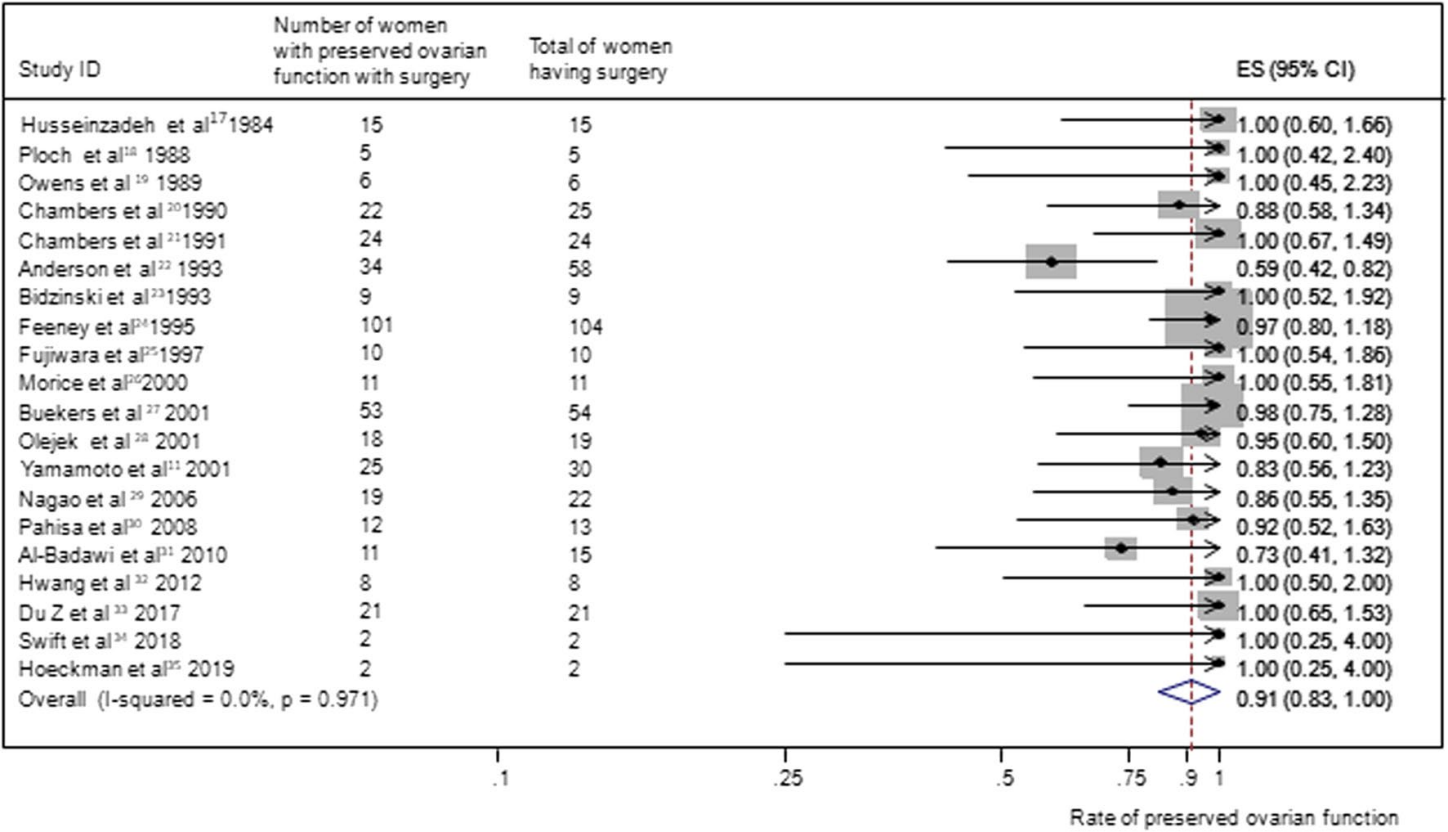
Ovarian preservation and surgery only group. Forest plot showing the proportions of cervical cancer patients (with 95% Confidence Intervals) with preserved ovarian function following ovarian transposition who had surgery alone

**Fig. 4:**
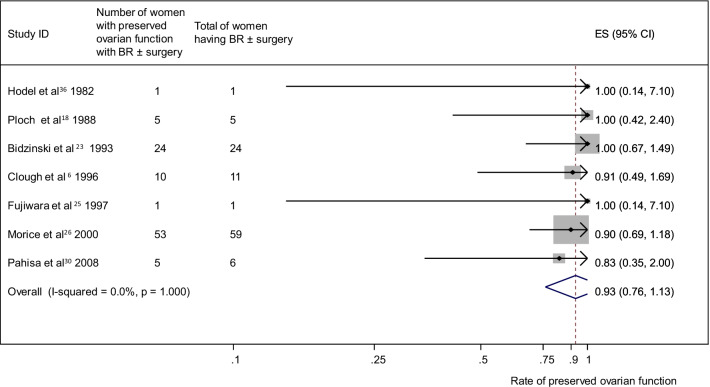
Ovarian preservation and brachytherapy (BR) ± surgery group. Forest plot showing the proportions of cervical cancer patients (with 95% Confidence Intervals) with preserved ovarian function following ovarian transposition who had brachytherapy (BR) ± surgery.

**Fig. 5:**
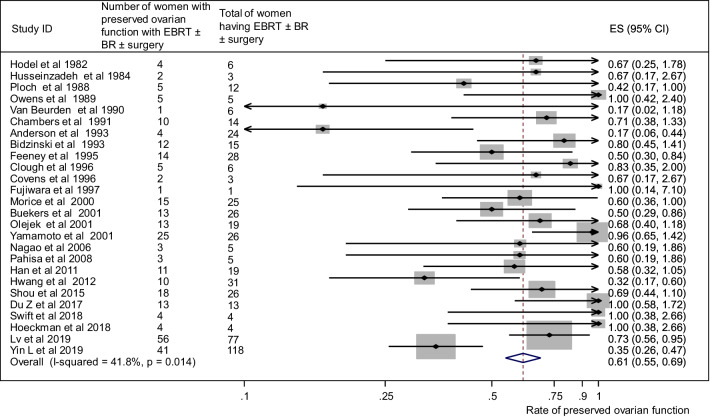
Ovarian preservation and external beam radiotherapy (EBRT) + surgery ± brachytherapy (BR) group. Forest plot showing the proportions of cervical cancer patients (with 95% Confidence Intervals) with preserved ovarian function following ovarian transposition who had external beam radiotherapy (EBRT) + surgery ± brachytherapy (BR)

In group A, results from 20 studies (11, 17–35) (n = 433 women) reporting ovarian function as an outcome gave a summary proportion of 91 percent (95% CI 83–100) for ovarian function preservation. No significant variation across the studies was observed (I2 = 0.0%, p = 0.97) (Fig. 3). The summary proportion from seven studies [6, 18, 23, 25, 26, 30, 36] (n = 107 women) reporting ovarian function preservation was 93% (95% CI 76–113) in group B. No significant variation across the studies was observed (I2 = 0.0%, p = 1.00) (Fig. 4). Pooled results from 26 studies [6, 11, 17–19, 21–32, 34–39] (n = 512 women) reporting ovarian function in group C rendered a summary proportion of 61% (95% CI 55–69) for ovarian function preservation. A significant variation across the studies was observed (I2 = 41.9, p = 0.014) (Fig. 5).

### No ovarian cyst formation

**Fig. 6:**
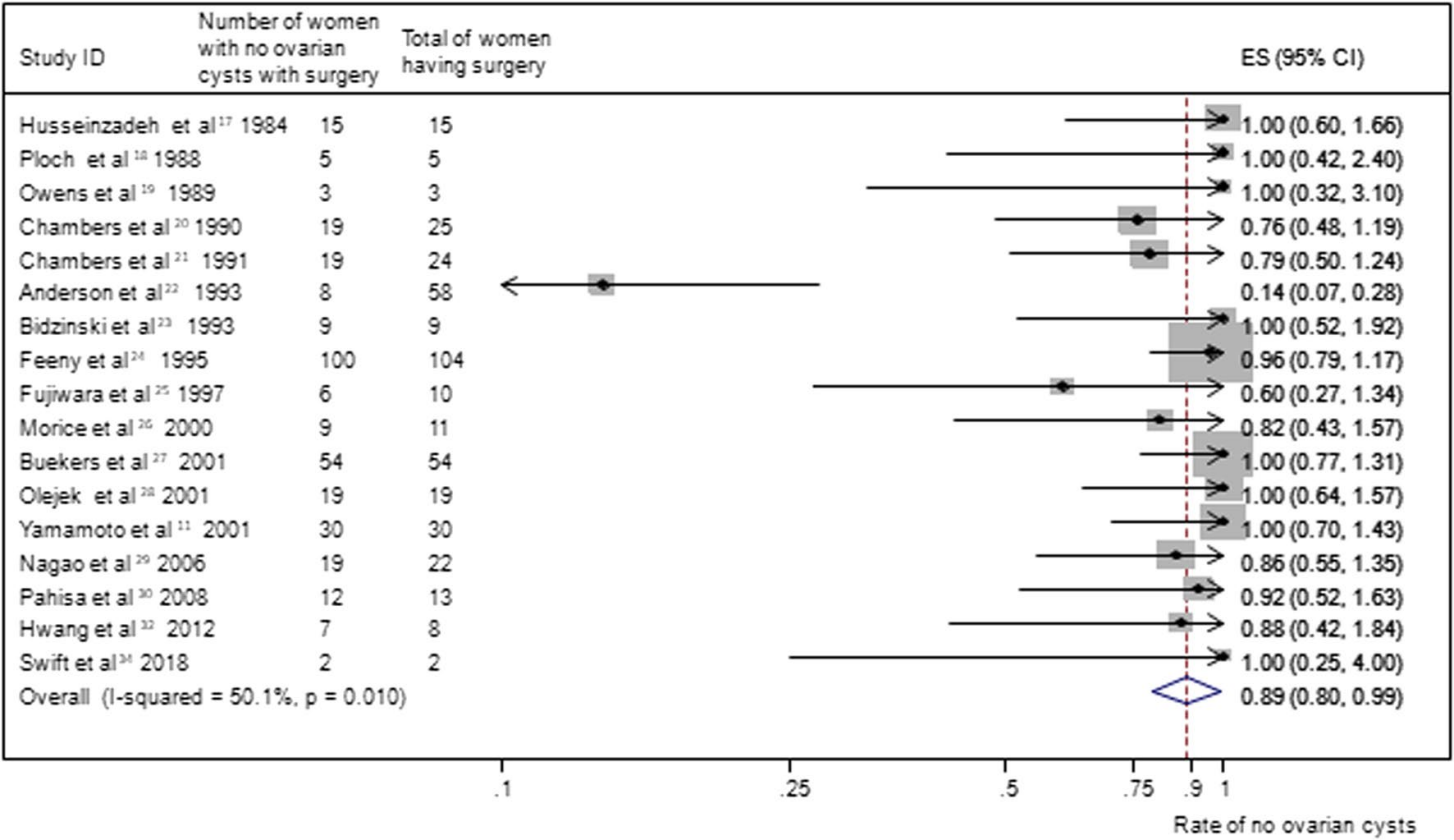
No ovarian cyst formation and surgery only group. Forest plot showing the proportions of cervical cancer patients (with 95% Confidence Intervals) who developed ovarian cysts following ovarian transposition who had surgery alone

**Fig. 7:**
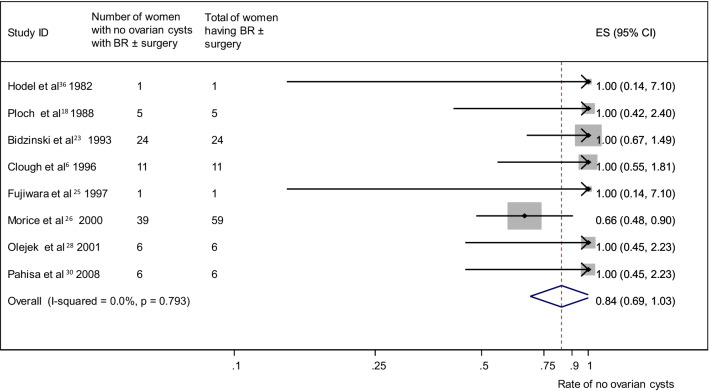
No ovarian cyst formation and brachytherapy (BR) ± surgery group. Forest plot showing the proportions of cervical cancer patients (with 95% Confidence Intervals) with no ovarian cyst formation following ovarian transposition who had brachytherapy (BR) ± surgery

**Fig. 8:**
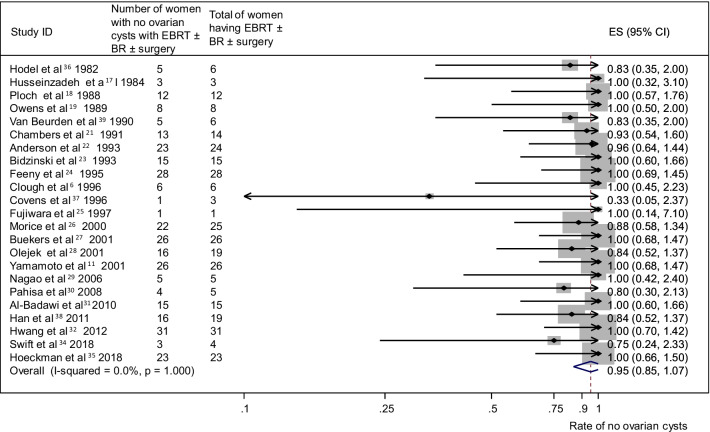
No ovarian cyst formation and external beam radiotherapy (EBRT) + surgery ± brachytherapy (BR) group. Forest plot showing the proportions of cervical cancer patients (with 95% Confidence Intervals) who developed ovarian cysts following ovarian transposition who had external beam radiotherapy (EBRT) + surgery ± brachytherapy (BR)

A pooled analysis of 17 studies [11, 17–30, 32, 34] (n = 392) reporting no ovarian cyst formation as an outcome in group A provided a summary proportion of 89% (95% CI 80–99) (Fig. 6). The variation across studies was significant (I2 = 50.1%, p = 0.01). Pooling data from eight studies [6, 18, 23, 25, 26, 28, 30, 36] (n = 113) reporting no ovarian cyst formation as an outcome in group B yielded a summary proportion of 84% (95% CI 69–103). There was no significant variation across the studies (I2 = 0.0%, p = 0.793) (Fig. 7). Pooled results from 23 studies [6, 17–19, 21–30, 32, 34–39] (n = 315) reporting no ovarian cyst formation in group C rendered a summary proportion of 95% for ovarian cyst formation (95% CI 85–107) with no significant variation across the studies (I2 = 0.0%, p = 1) (Fig. 8).

### No metastases to the transposed ovaries

Pooled results from 18 studies in group A reporting no metastases to the transposed ovaries rendered a summary proportion of 99% (95% CIs 91–108) for no metastases. No significant variation across the studies was observed (I2 = 0.0%, p = 1.00). Pooled results from seven studies in group B reporting no metastases to the transposed ovaries rendered a summary proportion of 99% (95% CIs 82–120) for no metastases. No significant variation across the studies was observed (I2 = 0.0%, p = 1.00). Only one study in group C reported an estimate of 96% (95% CI 64–144) for no ovarian metastases (22). In absolute numbers, all four studies reported 6/292 recurrences to the transposed ovaries (2%) [22, 24, 26, 43].

## Discussion

The evolution of OT procedures fostered a significant breakthrough in FP surgeries [44]. Ovarian transposition aims to maintain ovarian function in premenopausal women treated with pelvic RT [11]. That said, ovarian function preservation is critical being associated with decreased mortality in women younger than 50 years or those who never used oestrogen therapy, and at no age is oophorectomy associated with increased survival [45]. Oophoropexy is now established as a straightforward and reliable method with reduced morbidity [4]. The primary technique for transposing the ovaries has been previously described [46]. Published data show differences in functional outcomes such as ovarian failure, ovarian cysts, and metastases to the transposed ovaries. Earlier, we demonstrated the efficacy and safety of performing OT in women with gynaecological cancers [12]. Herein, we updated on our previous systematic review by specifically focusing on the outcomes of women with cervical cancer. Our systematic review of 29 studies confirms the concept that, in cervical cancer patients, OT can be offered as a specific treatment package, which is associated with high preservation of ovarian function, an expected rate of symptomatic ovarian cysts and very low risk of metastases in the transposed ovaries. To our knowledge, this is the first meta-analysis of the OT efficacy and safety in cervical cancer patients. In this update, the addition of studies published from 2014 to date did not alter the results published in our previous study [12], which further strengthens the impact of OT on the examined outcomes (Table 2).

**Table 2:** Comparison of the effect estimates on the desired outcomes between previous [12] and updated meta-analysis following addition of new studies for the selected early stage cervical cancer groups

Outcome/type of therapy	New studies included in the updated meta-analysis (author & year of publication)					
	Surgery	Effect Estimate with 95% CI 2021 vs 2014 [12]	Surgery ± BR	Effect Estimate with 95% CI 2021 vs 2014 [12]	Surgery ± BR ± EBRT	Effect Estimate with 95% CI 2021 vs 2014 [12]
Preserved Ovarian Function	Du Z [33] 2017 Hoeckman [35] 2018 Swift [34] 2018	**0.91 (0.83 to 1.00)** vs 0.91 (0.82 to 1.00)	None	No change	Du Z [33] 2017 Hoeckman [35] 2018 Lv [41] 2019 Shou [40] 2015 Swift [34] 2018 Yin L [42] 2019	**0.61 (0.55 to 0.69)** vs 0.62 (0.53 to 0.72)
Metastases	Zhao [43] 2013	**0.02 (0.01 to 0.05)** vs 0.02 (0.00 to 0.08)	None	No change	None	No change
Ovarian Cysts	None	No change	None	No change	Swift [34] 2018	**0.16 (0.10 to 0.27)** vs 0.16 (0.10 to 0.26)

In early cervical cancer, patient selection for ovarian reposition is challenging because it is difficult to decide who would require postoperative RT prior to the surgical procedure [47]. This problem was overcome by interrogation of three treatment groups by a single-arm meta-analysis: (a) those who had surgery only (Group A); patients who had postoperative BT (Group B); patients who had primary EBRT ± surgery ± BR (Group C). Ovarian transposition was the fixed variable for all groups. This approach allowed for an indirect comparison between surgery, BR and EBRT without the risk of increasing missing data. As OT does not protect against the detrimental effects of chemotherapy [48], a chemotherapy group was not included in the analysis. Ovarian survival may approach 70% when different chemotherapy types and doses of chemotherapy are used. Therefore, it would have been unlikely to draw meaningful conclusions regarding the effectiveness of OT in patients receiving both PR and chemotherapy, whereas ovarian survival appears to be further reduced [46].

**Fig. 9:**
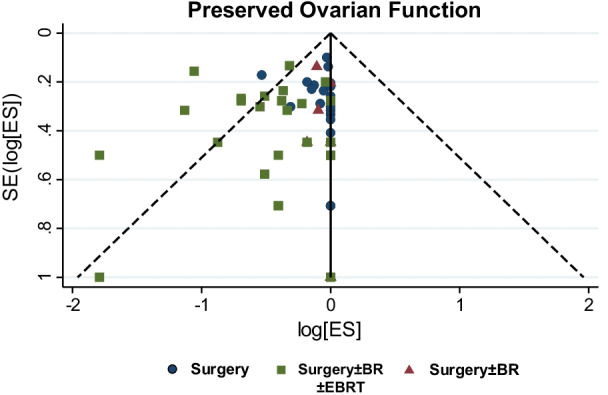
Funnel plot showing the probability of ovarian function preservation amongst the three groups of women with early stage cervical cancer. In group C (surgery ± BR ± EBRT), a significant variation was observed. Forcenull was applied forcing the vertical line at the centre of the funnel to be plotted at the null treatment effect of zero

In our studies, the ovarian function was assessed by patients' symptoms [23, 24, 26, 27, 30–32, 34, 35, 40, 41], serum FSH levels [11, 23–35, 37–42], E2 levels [11, 26, 28, 30, 33, 35, 38, 41, 42] and complemented by body temperature [11, 27], Progesterone [11], PRL [28] and Testosterone [28] to a lesser extent. This obvious variation, added to the differential study size, menopausal laboratory values and the diverse timing of hormone assessment could be potentially responsible for some study heterogeneity in relation to the ovarian survival (Fig. 9). In oncology patients, AMH can be serially measured to assess the impact of chemotoxic agents on ovarian function, to forecast future fertility and the onset of premature ovarian insufficiency [49]. Nevertheless, no test is highly accurate in predicting fertility potential. Various factors affect ovarian endocrine function, and many studies confirmed that RT administration following OT significantly affected ovarian function [50]. Radiotherapy and patient age remain the most important confounding factors [27], the mechanism being a dose- and age-related reduction in the ovarian follicular pool [51]. Increasing age (40 years and above) is associated with a decreasing ovarian reserve, which carries a higher risk for premature failure, even with OT [52]. The preservation of ovarian function is also related to its translocated position [53]. Despite the adoption of various OT techniques based on the treatment plan and pelvic anatomy [11], lateral transposition above the pelvic brim appears to be superior [10, 28, 31]. Evidence shows that transposition of the ovaries more than 1.5 cm above the iliac crest is associated with successful ovarian function preservation [32]. The transposed ovaries should have the same at-risk volume margins compared to normal ovaries to allow for potential transposed ovarian movement [54]. Dosimetry studies have demonstrated the superiority of intensity-modulated proton therapy (IMPT) compared with intensity-modulated radiation therapy [IMRT] in decreasing the integral dose to essential organs at risk in patients with gynaecologic malignancies [55]. In patients who received postoperative RT, the ovarian function was affected, suggesting that the standard ovarian limited dose used in IMRT disrupted ovarian function [33]. Lately, few women may prefer to carry pregnancy to term after cancer treatment. For this rather non-established concept, high-precision modern radiation therapy techniques for target volume delineation to spare dose to the unaffected uterus may allow uterine sparing chemoradiation [56]. Whether this may preserve fertility, adding to the ability to carry a pregnancy to term after cancer treatment without compromising cancer control is fully unclear. Nevertheless, the selection of younger patients and adequate dose limitation of the transposed ovary is required to maintain ovarian function [42].

The type of transposition and the position of transposed ovaries did not adversely affect the ovarian function for those patients who received no adjuvant RT, which was highly preserved. In our review, the proportion of patients, who received only surgery that became menopausal, was 10%, in contrast to studies showing 5% or less [49]. A plausible explanation can be the migration of the ovaries back to the radiation field, following their release from the fixation point; a finding seen at laparoscopy six months following OT [48]. Interestingly, the ovarian function was better preserved in those patients who had BR ± surgery compared with those patients who had surgery only. We speculate a "timing effect", whereas OT prior to irradiation as opposed to simultaneously at surgery does not allow for scar tissue formation [48]. Simultaneous transposition at the time of extensive surgery increases the risk for vascular compromise to the ovary from trauma or RH. Retroperitoneal ovarian tunnelling to prevent vascular torsion may be effective towards reducing the radiation dose to the ovarian vessels [57]. Nevertheless, as the incidence of ovarian failure appears to increase with the length of follow up -with 7% failing within three years and up to 50% within five years [19]-, based on our analysis, group B had a shorter mean follow up than group A, which may partly explain the better performance of group BR. Equally, approximately 67% of patients who had lost ovarian endocrine function three months after radiotherapy, regained it one-year post-RT [41]. It appears that mature follicles with hormone-secreting function are more sensitive to radiation than the primordial follicles [58]. We also observed that ovarian function was better preserved in those patients who have BR only without EBRT. Covens et al. thought that BR little harms the ovarian function following OT [37]. The ovarian vascular supply is more likely to be damaged by EBRT, as it loops down the pelvic brim before ascending again to the transposed position. Therefore, OT prior to EBRT warrants great care to position the pedicles in addition to the ovaries.

Cervical cancer is a non-hormone dependent tumour, and the probability of early cervical cancer metastasis to the ovary is extremely low. Quite disturbingly, two case reports have reported ovarian metastases in transposed ovaries [59, 60]. We demonstrated that, in line with the common consensus, the risk of ovarian carcinoma affecting the transposed ovaries is extremely low. This could be further reduced if opportunistic salpingectomy was performed during the surgical procedure [61]. Several risk factors have been identified for ovarian involvement [62]. In women with early-stage cervical adenocarcinoma, ovarian preservation has no effect on prognosis [63]. Ovarian relapse is unlikely to develop even after long observation periods [62, 63]. However, Sutton et al. reported a squamous cell carcinoma incidence of 0.5% compared to adenocarcinoma of 1.7% [64]. Nevertheless, OT should not be recommended in women with an inherited predisposition to ovarian cancer or malignancies at moderate-to-high risk of ovarian metastases [10]. In the four studies reporting ovarian metastases in the transposed ovaries, there was a balanced case mix of open and minimally invasive surgeries. Unfortunately, the exact numbers for both surgical groups were not available. In the post LACC trial era, this remains a sensitive topic [65]. 

On imaging, the transposed ovaries appear as ovoid structures with follicles adjacent to surgical clips [66]. They should not be confused with peritoneal implants. Benign functional or inclusions cysts should be easily distinguished from primary or secondary malignancies. The risk for developing symptomatic ovarian cysts following OT is higher than in the general population [67]. Risk factors for cyst development tend to relate to the surgical procedure, including extensive ovarian mobilisation or history of previous surgery, and gynaecological pathologies such as endometriosis or pelvic inflammatory disease [20]. Although this risk is multifactorial, ovarian function preservation makes the ovary intrinsically prone to developing functional cysts. Therefore, it is not surprising that more than 10% of patients in the surgery only group and in the BR ± surgery group were symptomatic for ovarian cysts. These were 5% in the EBRT ± surgery ± BR group, thus reflecting the ovarian status or in cases where surgery was not part of the treatment modality. Nevertheless, substantial heterogeneity was demonstrated in studies reporting ovarian cyst formation in the surgery only group, likely due to study size, different cyst detection imaging modalities, surveillance follow up protocols and duration of follow-up. Subcutaneous transposition may have potential benefits for early detection and more straightforward diagnosis of ovarian cysts, access to ovarian cyst removal and facilitation of in vitro fertilization [25]. If minimally invasive surgery induces less postoperative adhesions, a lower incidence of postoperative ovarian cysts should be expected. Although prolonged ovarian downregulation is initially required, frequently, a surgical procedure involving needle puncture, cystectomy, or oophorectomy is necessary. Nevertheless, it is unclear whether other intraoperative complications, such as haematoma or fallopian tube infarction, are encountered [22]. Transposition of only one ovary reduces the risk of developing functional ovarian cysts [27]. In all studies, the decision to transpose one ovary instead of two was due to the uncertainty of additional risks, such as cyst formation or torsion during surgery. Symptomatic cysts were detected by imaging, and either conservative or surgical treatment was used. In spite of the surgical clips attached to the ovaries being visible on CT scans, the appearance of the ovary does not reliably predict the development of complications [63]. While future research efforts will focus on direct comparisons of the incidence of ovarian cysts to the background risk of ovarian cysts between similar age groups, we acknowledge the challenges related to the frequency of diagnosing ovarian cysts. Incidental diagnosis is not uncommon; however symptomatic cysts requiring intervention prompt more frequent and prolonged follow-up to outrule ovarian metastases, all that suggesting a potential lag time effect in the diagnosis of ovarian cysts compared with the general population. The patients should be fully informed of all possible risks associated with ovarian reposition, including ovarian cyst formation.

Data from the MarketScan database reported a 8.2% prevalence of OT in women with cervical cancer [68]. The probability of performing OT was higher for women who underwent cancer-directed surgery prior to RT compared with women who underwent RT prior to cancer-directed surgery or no surgery at all. Advanced imaging including MRI, and occasionally PET-CT could be used to confirm patient eligibility for OT and exclude ovarian involvement [69]. In cervical cancer, where resources are available, MRI-based protocols can be tailored to the individual patient needs to assist with risk stratification and treatment design.

Strength of the study was the use of sound methodology and quality indicators in conducting the systematic literature review. The indirect comparison of the selected groups has eliminated the differential treatment strategy as a confounding factor. There was little or no evidence of publication bias in the three groups. The employment of the random-effects model enabled study variability control. As a limitation, we acknowledge that most studies were retrospective and non-comparative; they were all observational. Most of these studies were not designed for the specific outcomes examined, except for the ovarian function. The lack of clinical trials limits the data quality on the desired outcomes. Stratification of the results by confounding factors, such as age and follow-up was limited. Therefore, a certain level of clinical heterogeneity could be expected. Furthermore, we did not incorporate any survival data, which is important due to the implications of the extent of radiation outside the pelvic brim. We acknowledge that adjuvant BR alone is not the standard of care in the treatment of cervical cancer and may not be impactful on clinical decision making. Future work will attempt a sensitivity analysis of the BR subgroup within the expanded RT group. Furthermore, the variation in the types of performed OT surgeries can not be overlooked. While minimally invasive surgery is superior to open sugery to secure transposition of the ovaries a safe distance from the umbilicus [32], in the post LACC trial era, this may not be feasible. Future work will clarify whether type of surgery can be a determinant of ovarian survival following OT. In addition, the role of exogenous HRT alongside with reproductive outcomes should be further discussed [70]

No studies, including those published after 2018, employed the new FIGO 2018 classification, which currently provides the most accurate information pertaining to disease prognosis [71]. This would potentially complicate the discussion about ovarian metastases and stage designation. Reclassification according to the new FIGO 2018 staging scheme would potentially alter the summary proportions in the three groups. Future work will aim to examine the prognostic performance of the new FIGO classification added to the value of information about OT.

## Conclusions

This systematic review and secondary single-arm meta-analysis follows on from our previous work and confirms the efficacy and safety of OT in cervical cancer patients undergoing radio-surgical treatment. It achieves high preservation of ovarian function and carries a negligible risk of metastases to the transposed ovaries, despite a substantial incidence of symptomatic ovarian cysts. For the younger population, this is important information, as these women may prefer to carry pregnancy to term after cancer treatment, which would require modern radiation therapy approaches. In our study, the surgery alone group followed by the postoperative BR group performed best for the outcomes in question. Modern markers of ovarian reserve, such as AMH should be serially employed to monitor ovarian function. Larger prospective studies in cervical cancer patients undergoing OT with a longer follow-up time are warranted to clarify the predictors of ovarian function preservation. As the quality of care remains an important issue in the cancer trajectory, standardization of the OT procedure and multidisciplinary team involvement is required to fully evaluate the effectiveness of this relatively underutilized procedure.

## Data Availability

Data generated and analyzed during the current studies are not publicly available due to previous confidentiality concerns, but can be obtained from the corresponding author upon reasonable request.
